# mTOR Deletion Alleviates CD4+ T-Cell Dysfunction in Sepsis through Reducing CTLA4 Accumulation Mediated by Rescuing Autophagy

**DOI:** 10.1155/2024/4233439

**Published:** 2024-07-25

**Authors:** Xianli Lei, Guoyu Zhao, Yawen Xie, Na Cui

**Affiliations:** Department of Critical Care Medicine State Key Laboratory of Complex Severe and Rare Diseases Peking Union Medical College Hospital Chinese Academy of Medical Science and Peking Union Medical College, Beijing 100730, China

## Abstract

Sepsis has been the leading cause of death in ICU patients. CD4+ T cells are the mainstay of the body's immune system, and the depletion of CD4+ T cells in sepsis is of great concern. Cytotoxic T lymphocyte-associated protein 4 (CTLA4) is a negative immunomodulator for T cell activation and degradation through the autophagy-lysosome pathway. Mammalian target of rapamycin (mTOR) is the most classical upstream regulator of autophagy. With a mouse model of sepsis through cecal ligation and puncture (CLP), T cell specific-mTOR/tuberous sclerosis complex 1 (TSC1)-knockout mice, and bafilomycin A1, a specific autophagosome-lysosome (A-L) fusion inhibitor, we primarily proved that mTOR could modulate the expression and accumulation of CTLA4 by regulating the onset process of autophagy such as A-L fusion. Given such a regulatory relationship, targeting mTOR could provide new light to improve immune function in sepsis, and the prospect of using rapamycin in the clinic would be worth exploring further.

## 1. Introduction

Sepsis, defined as a life-threatening organ dysfunction caused by dysregulated host response to infection, remains a significant global health challenge [1]. Thus, it is urgent to find new treatment methods for sepsis. As a pathophysiological characteristic of sepsis, hyperinflammation, and immune suppression draws our attention [2]. In sepsis, CD4+ T-cell numbers, phenotypes, and functions are relatively different, which is the leading cause of immunosuppression [3]. There are multiple reasons for these results, such as depletion of CD4+ T cells by apoptosis and necrosis, and increased expression of negative regulatory factors, such as cytotoxic T lymphocyte-associated protein 4 (CTLA4), programed cell death protein 1 (PD-1), which negatively regulate proliferation and polarization [2]. Focusing on the expression and signaling networks of these negative regulatory molecules can definitely provide emerging directions for preserving CD4+ T cells for the treatment of sepsis.

CTLA4 is a representative of the immunoglobulin superfamily and, as a coinhibitory receptor, it binds with 20−50-fold higher affinity to B7 than CD28 [4, 5]. CTLA4 is expressed in regulatory and activated T cells and is mainly localized in the cytoplasm, acting on the cell surface. Endocytosis and lysosomal degradation are the processes of movement, recycling, and degradation between the cytoplasm and the cell surface for it [6]. Numerous studies have confirmed that CTLA4 is closely associated with progression and prognosis in sepsis [7, 8], but the pattern of expression, exact function, and the associated regulatory network remain unclear. Therefore, we proceeded to understand the role of CTLA4 in driving lymphocyte dysfunction during sepsis and the associated regulatory mechanisms.

**Figure 1:**
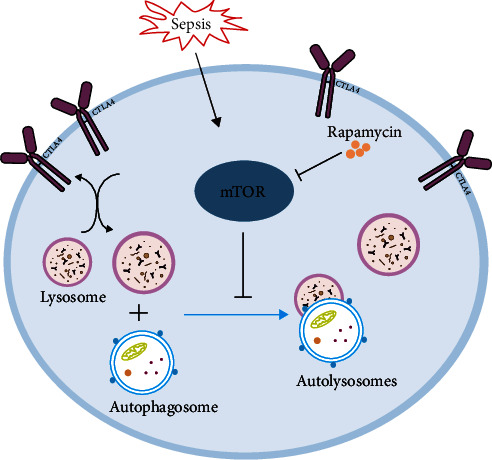
The graphical abstract of the work. The mTOR-autophagy-CTLA4 regulatory axis in sepsis. mTOR, mammalian target of rapamycin. CTLA, cytotoxic T lymphocyte-associated protein 4.

Autophagy is a recognized lysosomal degradation pathway, with major processes including cargo selection, autophagosome-lysosome fusion, and autophagic degradation [9]. Extensive studies have confirmed that autophagy is closely interrelated with T cell proliferation, activation, and immune function [10]. Gerner et al. [11] demonstrated the role of autophagy in regulating the expression of CD39, an important immune regulator, in regulatory T cells [11]. It has been documented that autophagy inhibitors chloroquine in murine skin and heart transplantation can prevent the degradation of CTLA4 [12]. Considering the regulatory role of CTLA4 on T cells and its lysosomal degradation pathway, we wanted to know the role of autophagy in CTLA4 function in sepsis. mTOR, a serine/threonine, regulates cell metabolism, differentiation, and death in response to various external stimuli [13]. Our previous studies demonstrated the regulatory role of mTOR on the autophagy of T cells [14]. Therefore, we speculated whether mTOR could regulate the degradation of CTLA4 through autophagy to control the function of CD4+ T cells. Therefore, we designed this experiment to explore the relationship between mTOR, autophagy, and CTLA4 in sepsis, the mechanism related was shown in the graphical abstract (Figure 1).

## 2. Methods and Materials

### 2.1. Mice and Model

Healthy male C57BL/6 N mice purchased from the Institute of Analysis and Testing, Beijing Academy of Science and Technology (Beijing, China), and aged 6–8 weeks and weighing 18–20 g were used in this study. As described previously, T cell-specific mTOR and TSC1 genetic modification mice were created [15]. Based on the method used, we created the cecal ligation and puncture (CLP) mouse model of sepsis [16]. First, mice were given an isoflurane inhalation for anesthesia. Next, a 1 cm midline incision was made on the lower abdomen, and the cecum was tied midway between the base and the distal pole. A 21-gauge needle was used to penetrate the cecal stump once, and some feces were carefully squeezed out. The cecum was placed back into its original intraabdominal location and the abdomen was sutured. Finally, each animal received 1 mL of saline subcutaneously for fluid resuscitation. A similar operation without CLP was performed on the mice in the sham group. One hour following the CLP procedure, intraperitoneal bafilomycin A1 (1 mg/kg; Solarbio, Beijing, China) was given. Rapamycin (6 mg/kg; Shanghai Yuanye Bio-Technology, Shanghai, China) was administered intraperitoneally 3 hr after the CLP operation. Eventually, the mice were placed in cages with enough food and water.

### 2.2. Lymphocyte Isolation and Cell Sorting

Mice were euthanized 12 hr after surgery, and spleens were removed and stored in PBS solution. Spleens were pressed gently with glass slides. The resulting homogenates were washed with phosphate-buffered saline and red blood cells were lysed by ammonium–chloride–potassium lysis buffer. Splenocytes that had been centrifuged were again suspended in RPMI-1640 media (Sigma–Aldrich, St. Louis, MO, USA). After 30 min of staining on ice with biotin-conjugated anti-CD4 antibody, splenocytes were rinsed, treated for 15 min with magnetic streptavidin beads, and then reassembled in cell-sorting buffer. Using distinct columns and negative selection, CD4+ T cells were separated. The purity of the resulting CD4 + T cells was determined as >90% assured by FACS detection. The sorted cells were counted and subsequently used for the next step of the experiment.

### 2.3. Transmission Electron Microscopy

Transmission electron microscopy (TEM) is used to observe the morphological changes in the onset and development of autophagy. CD4+ T cells selected from each group (WT, WT + CLP, mTOR-CKO + CLP, TSC1-CKO + CLP, and so on) were collected, and the cell suspension was centrifuged, washed, and fixed by 2.5% glutaraldehyde. The collected CD4+ T cells were then washed with sodium phosphate buffer (0.1 M, pH 7.2), shock-fixed with 1% OsO4 for 3 hr, dehydrated with ethanol, and finally embedded with Spurr resin. After stained with aqueous uranyl acetate and lead citrate, we get the ultrathin sections using an ultramicrotome (Leica EM UC6, German). We observed the ultrastructure of autophagosomes and autolysosomes by a transmission electron microscope (JEM1230; Jeol, Tokyo, Japan).

### 2.4. Western Blotting

Total protein of spleen CD4+ T cells was prepared with RIPA buffer and the concentration was measured using the bicinchoninic acid method. Approximately 30 *μ*g of protein samples were added to each well of SDS-PAGE. After membrane transfer, electrophoresis, blocking with 5% milk, and lastly an overnight incubation with a particular primary antibody. Three rounds of washing the membrane with tris-buffered saline with 0.1% Tween-20 were conducted. The membrane was incubated with the corresponding horseradish-peroxidase-conjugated secondary antibody for 1 hr. Finally, A Bio-Rad ChemiDoc XRS + device (Bio-Rad, Hercules, CA, USA) was used to take the images, and QuantityOne software (Bio-Rad) was used to conduct densitometric analysis. The following antibodies (all from Affinity Biosciences, Cincinnati, OH, USA) were used for western blotting: mTOR antibody, Phospho-mTOR (Ser2448) antibody, Phospho-p70 S6 Kinase (Thr389/Thr412) antibody, SQSTM1/p62 antibody, LC3A/B antibody, IL1 beta antibody, and the rest antibodies will supplied as required.

### 2.5. Statistical Analysis

Version 9.0 of GraphPad Prism was utilized for data processing and graphing. For continuously dispersed data that were normally distributed, means ± standard deviations (SDs) were shown. Analysis of variance was used to evaluate differences, and then the test for the least significant difference was performed. It was deemed statistically significant when *P*  < 0.05.

## 3. Result

### 3.1. CD4+ T Cells in Septic Mice Showed Decreased Ability in Proliferate and Cytokines Production

**Figure 2:**
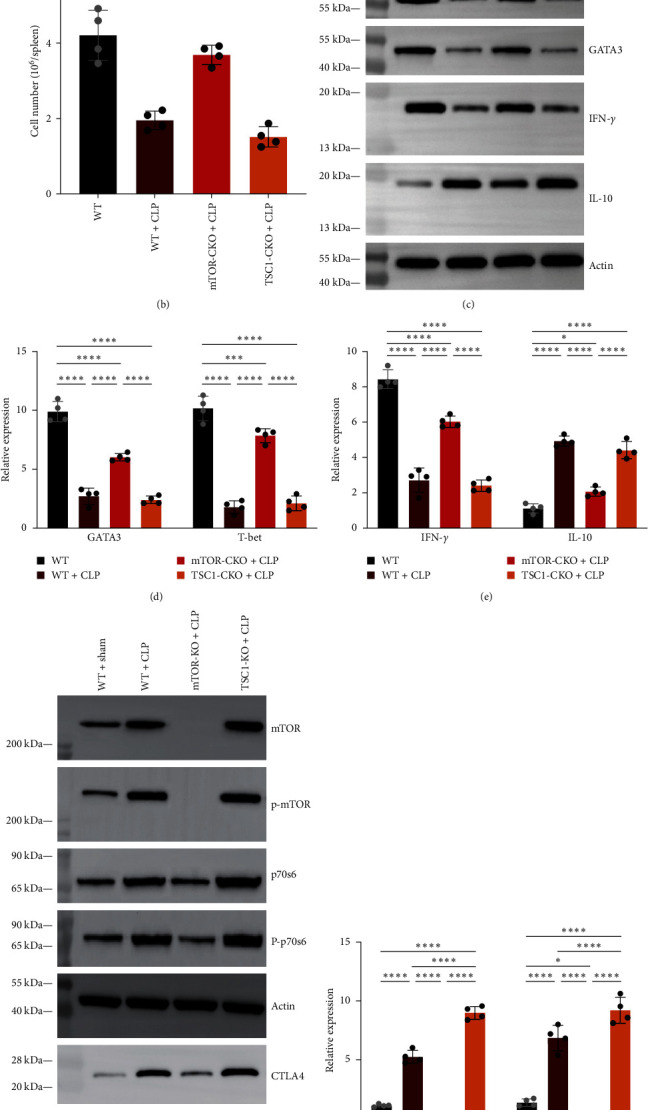
CD4+ T cells turn dysfunctional, with elevated expression of mTOR and CTLA4 in sepsis. (a and b) Sorting and counting CD4+ T cells with flow cytometry. (c–h) The protein levels of T-bet, GATA3, INF-*γ*, IL-10, phospho (p)-mTOR, p-P70 S6 kinase (S6K), and CTLA4 in CD4+ T cells were measured using western blotting in four different mouse models. The amount of each protein level was normalized to that of *β*-actin. *n* = 4 (healthy male C57BL/6 N mice and aged 6–8 weeks) biologically independent experiments. Means ± standard deviations (SDs) of four mice per group are shown. Analysis of variance was used to evaluate differences, and then the test for the least significant difference was performed. It was deemed statistically significant when *P*  < 0.05.  ^*∗*^*P* < 0.05,  ^*∗∗*^*P* < 0.01,  ^*∗∗∗*^*P* < 0.001,  ^*∗∗∗∗*^*P* < 0.0001.

To comprehend the response of CD4+ T cells in septic mice, we utilized flow cytometry and western blotting for cell counts and quantification of relevant components, respectively. The outcomes are illustrated in Figure 2. As the findings indicate that the CLP group had considerably less CD4+ T cell counts than the sham group (Figures 2(a) and 2(b)). We further assessed the expression levels of T-bet and GATA3, which are closely related to the proliferative ability of T cells. Compared to the sham group, the T-bet and GATA3 expression were considerably lower in the CLP group. Additionally, we examined the functional alterations of CD4+ T cells in septic mice and demonstrated a considerable downregulation of the pro-inflammatory factor INF-*γ* and a large elevation of the factor that suppresses inflammation, IL-10 (Figures 2(c), 2(d), and 2(e)). INF-*γ* promotes the inflammatory response during sepsis, but INF-*γ* production is dampened during sepsis. The mentioned findings indicate that aberrant CD4+ T cell proliferation and inflammation-related dysregulation occurred during sepsis.

### 3.2. Accumulated CTLA4 Was Observed in CD4+ T Cells of Septic Mice and Accompanied by Elevated mTOR Pathway Activity and Impaired Autophagy

**Figure 3:**
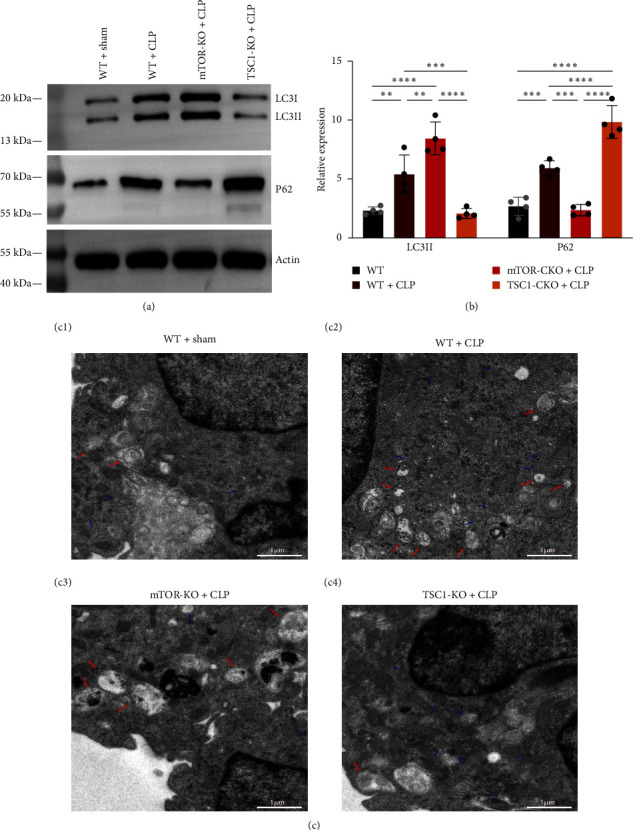
Autophagy in sepsis is modulated by mTOR. (a and b) Protein levels of LC3II/I and p62/sequestome 1 (SQSTM1) in CD4 + T cells were quantified by western blotting. The amount of each protein level was normalized to that of *β*-actin. Means ± standard deviations (SDs) of four mice per group are shown.  ^*∗∗*^*P* < 0.01,  ^*∗∗∗*^*P* < 0.001,  ^*∗∗∗∗*^*P* < 0.0001. (c) Morphology of autophagy in CD4 + T cells was investigated by transmission electron microscopy (TEM). Autophagosomes were double-membrane vacuoles containing cytosol or organelles. Autolysosomes were single-membrane structures containing digested cytoplasmic components. (c1) The normal morphologies of CD4+ T cells in control mice indicated a baseline autophagy state. (c2) Although autophagic vacuolization was enhanced in WT + CLP mice, there was no discernible increase in the number of autolysosomes. There were large autolysosomes with a lot of substances. (c3) Lck-mTOR + CLP mice showed more autophagic vacuolization and more autolysosomes. (c4) There were less autophagosomes and autolysosomes in Lck-TSC1+CLP mice. *n* = 4 (healthy male C57BL/6 N mice and aged 6–8 weeks) biologically independent experiments. Analysis of variance was used to evaluate differences, and then the test for the least significant difference was performed. It was deemed statistically significant when *P*  < 0.05.  ^*∗∗*^*P* < 0.01,  ^*∗∗∗*^*P* < 0.001,  ^*∗∗∗∗*^*P* < 0.0001.

Cytotoxic T lymphocyte-associated antigen 4 (CTLA4) acts as an immunonegative regulator, transmitting inhibitory signals to activated T cells. In order to elucidate the function of CTLA4 in CD4+ T cell dysfunction during sepsis, we employed western blotting to quantify CTLA4 expression, which revealed that CTLA4 was considerably accumulated in septic mice (Figure 2(h)). It has been demonstrated by numerous investigations that CTLA4 turnover and breakdown in T cells is lysosome-dependent and autophagy inhibitors can influence this process [12]. To explore the relationship of autophagy and the accumulation of CTLA4, we analyzed the concentrations of the autophagy proteins microtubule-associated protein LC3II/I and p62/SQSTM1 in septic and sham mice (Figures 3(a) and 3(b)). According to our findings, septic mice had a much larger proportion of LC3II/I in their CD4+ T cells than sham animals. Additionally, septic mice had greater levels of p62/SQSTM1. Based on the aforementioned event, autophagy appears to be triggered in septic mice, but it is disrupted during the breakdown phase. The TEM data also corroborated this conclusion. The phosphorylated mTOR and p70S6K concentrations, which indicate the activity of the mTOR pathway, were shown to be higher in septic mice (Figures 2(g) and 2(h)). The findings above imply that CTLA4 accumulation is accompanied by impaired autophagy and increased mTOR signaling pathway activity, there may be a regulatory link between mTOR, autophagy, and CTLA4 accumulation that impacts CD4+ T cell manifestation in sepsis.

### 3.3. mTOR Deletion Alleviated Autophagy Disorder and CTLA4 Accumulation in Septic Mice

Drawing from two distinct knockout models, namely T-cell-specific TSC1 knockout (TSC1-KO) mice and T-cell-specific mTOR knockout (mTOR-KO) mice, we investigated the mTOR pathway's regulatory function on the autophagy level of CD4+ T cells and CTLA4 accumulation in sepsis (Figures 3(a) and 3(b)). When comparing Lck-mTOR+CLP animals to wild-type (WT) + CLP mice, LC3 II/I expression was considerably higher and p62/SQSTM1 expression was reduced in CD4+ T cells. Compared to WT + CLP mice, TSC1-KO + CLP animals had increased levels of p62/SQSTM1 but decreased expression of LC3 II/I. This finding implies that mTOR pathway restriction may lessen the impairment of autophagy in septic mice and overactivation of the mTOR pathway can impair the autophagic process, maybe including the initiation phase and autophagosome-lysosome fusion. Furthermore, using the aforementioned models as a basis, we investigated the impact of mTOR depletion on CTLA4 accumulation in septic mice (Figure 2(h)). Compared to the WT + CLP group, CTLA4 expression was considerably lower in mTOR-KO + CLP mice. In TSC1-KO + CLP mice, however, CTLA4 expression was greater. Therefore, we demonstrated that mTOR depletion diminished CTLA4 accumulation in CD4+ T cells while improving autophagy deficiency in septic mice. Therefore, it is worth investigating further if the regulation of autophagy is responsible for the regulation of CTLA4 accumulation by mTOR.

### 3.4. The Impact of mTOR Deletion on CTLA4 Accumulation of CD4+ T Cells Can be Reversed by bafilomycinA1

**Figure 4:**
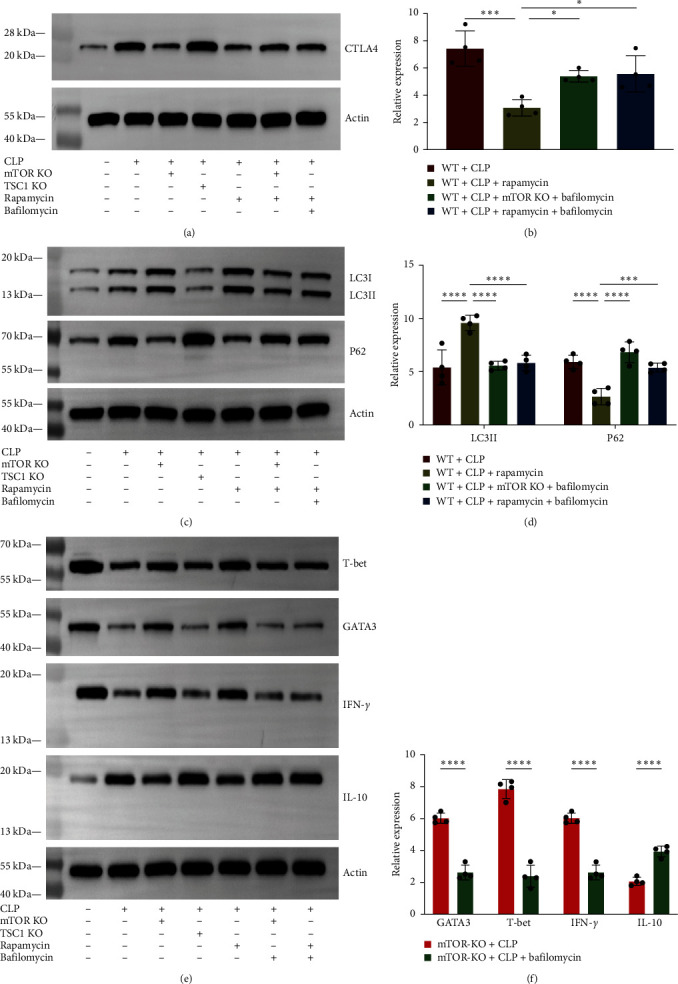
mTOR deletion restores CD4+ T-cell dysfunction in sepsis through alleviating CTLA4 accumulation and autophagy disorder. (a–d) The protein levels of CTLA4, LC3II/I, and p62/sequestome 1 (SQSTM1) in CD4+ T cells were measured using western blotting. (e and f) The protein levels of T-bet, GATA3, INF-*γ*, and IL-10 were measured using western blotting in the mice treated with or without bafilomycin A1. *n* = 4 (healthy male C57BL/6 N mice and aged 6–8 weeks) biologically independent experiments. Analysis of variance was used to evaluate differences, and then the test for the least significant difference was performed. It was deemed statistically significant when *P*  < 0.05.  ^*∗*^*P* < 0.05,  ^*∗∗∗*^*P* < 0.001,  ^*∗∗∗∗*^*P* < 0.0001.

Based on the previously mentioned regulatory effects of mTOR on autophagy and CTLA4 expression, we would like to further explore the relationship between the three even further. Bafilomycin A1, a V-ATPase inhibitor, is frequently employed as a specific autophagosome-lysosome fusion suppressor [17]. To specifically prevent autophagosome-lysosome fusion, bafilomycin A1 was utilized. CTLA4 expression of CD4+ T cells was considerably increased in mTOR-KO + CLP mice treated with bafilomycin A1 than mice not been treated with bafilomycin A1 (Figure 4(a)), and lower compared with mice in the WT + CLP + bafilomycin A1 group. This finding implies that the inhibitory effect of mTOR deletion on CTLA4 accumulation in CD4+ T cells was lessened by bafilomycin A1.

### 3.5. mTOR Deletion Restores CD4+ T-Cell Dysfunction in Sepsis through Alleviating CTLA4 Accumulation and Autophagy Disorder

In addition, our results showed that mTOR deletion ameliorated the proliferative suppression of T cells (indicators: T-bet and GATA3), and impaired cytokine production (indicators: INF-*γ* and IL-10), which we initially observed in septic mice (Figures 2(c), 2(d), and 2(e)). Compared with mice of mTOR-KO + CLP group, mTOR-KO + CLP mice treated with bafilomycin A1 showed that T-bet and GATA3 expression was significantly reduced, likewise INF-*γ* expression was further decreased, while IL-10 showed an elevated trend (Figures 4(e) and 4(f)). Above results indicated that the application of bafilomycin A1 reversed the regulation of CTLA4 accumulation by mTOR knockdown while also reversing the effect of mTOR deletion on T cell proliferation, cytokine production. As numerous studies have proved the regulation of T cell proliferation, activation, and related function are fundamental signals delivered by CTLA4 [18, 19], and its role in sepsis naturally cannot be ignored. Then, we could draw a conclusion that mTOR deletion restore CD4+ T-cell dysfunction in sepsis through alleviating CTLA4 accumulation by easing autophagy disorder.

### 3.6. Rapamycin Could Exert Modulatory Effects Similar to Those of mTOR Deletion on Autophagy and CTLA4 in Septic Mice

To test and explore the viability of targeting mTOR to regulate CD4+ T-cell autophagy in sepsis as a way to minimize CTLA4 accumulation and hence alleviate CD4+ T-cell dysfunction, we employed rapamycin, a particular mTOR inhibitor, to corroborate the genetic approach's results. When rapamycin was administered to WT + CLP mice, autophagy deficiencies were improved in comparison to WT + CLP mice, and there was also a notable decrease in CTLA4 expression, which is in line with the regulatory impact of mTOR knockdown (Figures 4(a), 4(b), 4(c), and 4(d)). In WT + CLP mice treated with both rapamycin and bafilomycin A1, we discovered that rapamycin's protective effect on autophagy and attenuating effect on CTLA4 accumulation were diminished, confirming that autophagy is involved in the regulation of CTLA4 accumulation by mTOR (Figures 4(a), 4(b), 4(c), and 4(d)). The above results shed new light on targeting mTOR to regulate the immune function of CD4+ T cells in sepsis.

## 4. Discussion

Sepsis is a multiorgan damage caused by dissonant immune response of infection, and the immunosuppression that occurs immediately is an important reason for the occurrence of secondary infections and increased associated mortality [20]. Immunosuppression is reflected in the suppression of immune cell function and a decrease in their number. Our results suggest that CTLA interfering with TCR costimulation activation, is closely associated with T cells drop in number and dysfunction, and this is in line with a recent study showing that CTLA4 is a highly essential regulator of T cell activity [21]. Additionally, we further exploited the associated regulatory pathways of CTLA4 accumulation in sepsis, and it was found that there was a close relationship between the mTOR signaling pathway, autophagy, and CTLA4 accumulation, and elucidated the mTOR-autophagy-CTLA4 regulatory axis. We finally provide that mTOR may be a potential target for sepsis treatment by alleviating autophagy disorder to reduce CTLA4 accumulation.

It seemed well-known that the number, proliferation, differentiation, and secretion of T cells in sepsis have been impaired. By virtue of flow cytometry, we observed a significant reduction in the number of CD4+ T cells and a markedly decreased proliferative capacity in CLP mice. Further analysis also revealed diminished levels of proliferation-related marker molecules, T-bet, and GATA3 expression compared to the WT group. Numerous reports have demonstrated that T-bet and GATA3 are critical T cell differentiation and proliferation regulators [22, 23]. The above results are consistent with the signs of T-cell failure reported in previous studies [24]. On the other hand, T cell damage was also manifested by impaired production of cytokines, including pro-inflammatory factors and anti-inflammatory factors [25]. In the present study, we found that the expression of anti-inflammatory cytokine IL-10 was elevated, while the expression levels of pro-inflammatory cytokines INF-*γ* were reduced in the CLP group. The above results suggest that the physiology of CD4+ T cells is disturbed in sepsis, as evidenced by the reduced number, impaired proliferation, and immune dysfunction. Meanwhile, we also observed that the expression of the negative regulator CTLA4 was enhanced in the CD4+ T cells of CLP mice. In line with previous studies, it was demonstrated that CTLA-4 expression is upregulated on CD4+ in sepsis [19, 26]. Anti-CTLA4 has been shown to have the effect of enhancing T-cell response and promoting INF-*γ* secretion [27]. CTLA-4 antibody treatment attenuates sepsis-induced cell death, preserves numbers, and improves survival in CLP sepsis models [7, 28]. In summary, the poor outcome of sepsis is associated with the physiological imbalance of T cells, and CTLA4 plays an important role in this and is a potential regulatory target. Therefore, it is extremely important to explore the CTLA4-associated CD4+ T regulatory network.

Under the regulation of two ligands, CD80 and CD86, CTLA4 either accumulates in endosomes and lysosomes or circulates to the cell surface via transendocytosis to exert inhibitory effects [29]. According to a recent study, CTLA4 degradation is inhibited by chloroquine whereas it is promoted by the proteasome inhibitor MG-132. The results above indicate that CTLA4 is destroyed via an autophagic-lysosomal route [12]. Our results suggested that the levels of autophagy-related markers LC3II/I and p62 were significantly changed in CD4+ T cells of septic mice, and we also observed incomplete autophagic flow and abnormal autophagosome morphology by TEM. The above findings are consistent with our previous study, indicating that CD4+ T cells in septic mice do have impaired autophagic flow [14], same as other studies [30]. We can further find that the trend of elevated CTLA4 is consistent with the level of autophagy impairment. As for the exact link between these two needs to be ascertained still.

Known as a modulator of autophagy, mTOR mediates multiple aspects of the autophagic process, such as initiation, process, and termination, by affecting the activity of critical autophagy-related genes [31]. Among them, the role of mTOR in regulating autophagosome-lysosome fusion during autophagy is gradually attracting attention [32, 33]. Similar regulatory effects of mTOR in sepsis were demonstrated in our previous studies [14]. In the current study, we observed that with the knockdown of TSC1, the autophagic flow was significantly impaired, and autophagy-related markers were also disrupted compared to the CLP group. Meanwhile, the accumulation of CTLA4 was more pronounced in the TSC1 knockout group. The opposite result was shown in the mTOR knockout mice. So does the regulation of autophagic flow by mTOR affect the degradation of CTLA4, we further tested our hypothesis with bafilomycin A1, a specific inhibitor of A-L fusion. In mTOR-KO + CLP mice treated with bafilomycin A1, the impaired autophagic flow was indeed evident, accompanied by a more pronounced accumulation of CTLA4 and CD4+ T dysfunction compared to mTOR-KO + CLP mice treated with saline. And we further proved that the expression of indicators for T cell proliferation and function is closely related to CTLA4. Putting the above results together, we draw a conclusion that mTOR can exacerbate the poor outcome of sepsis by inhibiting autophagosome-lysosome fusion, leading to impaired autophagy and further causing CTLA4 accumulation and exacerbating CD4+ T cell dysfunction in sepsis. Thus, we got a possible target for drug intervention, further supporting that targeting mTOR may be a new light for sepsis treatment.

It has previously demonstrated that rapamycin-treated CLP mice had considerably reduced sepsis-related cognitive impairments, which may be linked to rapamycin-induced autophagy, although further pathways have yet to be identified [34]. As a further complement, we further investigated the contribution of the mTOR inhibitor rapamycin on degradation of CTLA4. In mTOR overexpressing mice treated with rapamycin for 3 hr after CLP, we found that rapamycin inhibition of mTOR did attenuate CTLA4 accumulation and rescue CD4+ T cell deficiency and dysfunction, and the autophagy inhibitor bafilomycin A1 can reverse the above mitigating effects. Our results indicated that mTOR induced the dysfunction of autophagy contributing to the accumulation of CTLA4 and CD4+ T cells dysfunction, a process mitigated by mTOR inhibition in line with the previous study [35]. However, rapamycin did not affect the degradation of CTLA4 in mouse skin and heart transplantation models, standing in the opposite direction of our study [12]. A different study related to the survival of mouse cardiac allograft shows that the inhibit of mTOR signaling drives the expression of CTLA4 via blockade of Interferon regulatory factor 4 (IRF4) [36]. As Huang et al. [37] recently have proved the involvement of mTOR in a positive feedback loop between Rab4A, and Rab4A impacts cellular function through endocytic recycling which increased the expression of surface receptors CTLA-4 (CD152) [37]. This discrepancy might be accounted for differences in immune settings of sepsis, allograft, and systemic lupus erythematosus (SLE). We propose that autophagy serves as an important link in mTOR-CTLA4 accumulation axis, and this regulatory network can be further refined with future studies on the detailed mechanisms such as the specific autophagy molecules and the potential regular molecules such as Rab4A.

In summary, we may have found a contribution of the mTOR-autophagy-CTLA4 axis in sepsis-associated CD4+ T cell deficiency and dysfunction. Targeting mTOR, such as rapamycin, to reduce the accumulation of CTLA4 may represent a credible immunotherapeutic strategy aimed at restoring adaptive immune responses against microbial pathogens. But there are still some shortcomings in this study. First, it has been demonstrated that only CTLA4 on the cell surface has a role, but only the total CTLA4 expression in the cells was examined in this experiment. Although the related texts mention that total expression can also represent the function of CTLA4 [12]. Second, as we mentioned before, CTLA4 is essential for Treg development and function, previous study proved rapamycin treatment expanded CD4+CD25+FOXP3+ Tregs by inducing autophagy and correcting expression of CTLA-4 [38]. In our study, based on the overall immune environment in sepsis, we first explored the effect of mTOR regulation of CTLA4 on overall CD4+ T-cell function, and further experiments and discussions are needed in the future on the function of different cell subtypes, such as Treg cells, Th17 [39]. Importantly, what is described here about how mTOR interferes with CTLA4 through autophagy provides new insights to continue these studies. The eventual goal is to develop new treatments for patients with sepsis by continuing these studies.

## Data Availability

The corresponding author will provide the experimental data obtained and analyzed in this study upon request.
